# Specialist care and mental health is associated with long-term control of atopic dermatitis symptoms: A cross-sectional survey of patients and caregivers in the United States

**DOI:** 10.1016/j.jdin.2023.08.016

**Published:** 2023-08-31

**Authors:** Albert C. Chong, Alan Schwartz, Wendy Smith Begolka, Kathryn Z. Tullos, Korey Capozza

**Affiliations:** a Keck School of Medicine, University of Southern California, Los Angeles, California; b Department of Medical Education and Department of Pediatrics, University of Illinois Chicago, Chicago, Illinois; c National Eczema Association, Novato, California; d International Topical Steroid Awareness Network, Dacula, Georgia; e Global Parents for Eczema Research, Santa Barbara, California

**Keywords:** atopic dermatitis, mental health, specialist, United States

*To the Editor:* Atopic dermatitis (AD) affects 16.5% of children and 7.3% of adults in the United States, resulting in negative physical, mental, social, and financial consequences.^1^, ^2^, ^3^ In our recent survey of patient and caregiver experiences with AD across 8 high-income countries, pooled analysis linked specialist care to superior long-term control of symptoms.^3^ Meanwhile, health-related quality of life as measured by the EuroQol 5-dimensional (EQ-5D) was largely driven by anxiety/depression.^3^ In this subgroup study on US respondents, we sought to assess if specialist care is associated with better long-term control of symptoms and to identify factors related to anxiety/depression in AD since current insight on mental health care in AD is limited.

Data from US respondents were extracted from our previous institutional review board–approved survey.^3^ We fitted logistic regression models to provider type (generalist or specialist) for adult and child patients. We also fitted a linear regression model to the EQ-5D anxiety/depression dimension rating for adult patients. Independent variables included patient age, gender, AD severity, long-term control of symptoms score, and EQ-5D anxiety/depression rating (provider type model only). US respondents included 1293 adult patients with AD and 370 caregivers of children with AD (Table I). Specialist care for AD was significantly more frequent among adults (72%) vs children (66%). For adult patients, specialist care was independently associated with greater symptom control, greater AD severity, higher (worse) anxiety/depression rating, younger age, and male gender (Table II). Higher anxiety/depression rating was independently associated with decreased symptom control, increased AD severity, and nonmale gender.

**Table I:** Patient demographics

Variable	Child patients (as reported by their caregivers) (*N* = 370)	Adult patients (*N* = 1293)	*P* value
Patient age, y∗			<.001
Missing responses	102	42	
Mean (SD)	7.9 (5.0)	31.0 (8.8)	
Range	1-26	18-85	
Patient gender, *n* (%)			.583
Male	201 (54%)	668 (52%)	
Female	169 (46%)	624 (48%)	
Other	0 (0%)	1 (0%)	
AD provider, *n* (%)			<.001 (comparing specific provider types); .017 (specialists vs generalists)
Specialists			
Dermatologist	165 (45%)	798 (62%)	
Allergist	62 (17%)	98 (8%)	
Dermatology nurse	14 (4%)	29 (2%)	
Generalists			
Pediatrician	97 (26%)	153 (12%)	
Primary care / general practitioner	27 (7%)	197 (15%)	
Nurse practitioner	2 (1%)	8 (1%)	
Other (not classified as generalist or specialist)	3 (1%)	10 (1%)	
AD severity, *n* (%)			.012
Clear	24 (7%)	143 (11%)	
Mild	157 (42%)	469 (36%)	
Moderate	135 (37%)	520 (40%)	
Severe	54 (15%)	161 (13%)	
Long-term control of symptoms score, 0-100			<.001
Mean (SD)	57.1 (23.0)	50.9 (19.5)	
EQ-5D anxiety/depression rating, 1-5 (adults only)			
Missing responses		39	
Mean (SD)		2.3 (1.0)	

*AD*, Atopic dermatitis; *EQ-5D*, EuroQol 5-dimensional.

∗ Data from respondents who did not provide an interpretable age was included in the analyses. Caregivers provided their child’s age; 14 caregivers reported on children aged 18 to 26.

**Table II:** Regression models for provider type and anxiety/depression score

Predictors	Specialist provider (adult patients)			EQ-5D anxiety/depression rating (adult patients)			Specialist provider (child patients)		
	Odds ratio	CI	*P*	B	CI	*P*	Odds Ratio	CI	*P*
Long-term control of symptoms score	1.03	1.02-1.04	<.001	−0.02	−0.02 to−0.02	<.001	1.00	0.98-1.01	.85
AD severity									
Mild vs clear	1.58	1.29-1.94	<.001	0.04	−0.04 to 0.12	.36	1.40	0.67-2.81	.35
Moderate vs mild/clear	1.27	1.15-1.41	<.001	0.08	0.04-0.12	<.001	1.14	0.85-1.53	.37
Severe vs moderate/mild/clear	1.70	1.45-2.01	<.001	0.16	0.12-0.21	<.001	0.89	0.69-1.13	.33
EQ-5D anxiety/depression rating	1.34	1.14-1.57	<.001						
Patient age	0.97	0.95-0.98	<.001	0.00	−0.01 to 0.00	.34	1.03	0.98-1.10	.23
Patient male gender	1.46	1.11-1.91	.006	−0.17	−0.27 to −0.07	.001	1.32	0.78-2.25	.30
Observations	1204			1213			266		
R^2^	0.11			0.33			0.02		

*AD*, Atopic dermatitis; *EQ-5D*, EuroQol 5-dimensional.

This study yielded 2 key findings. First, US specialist care for adult patients with AD was associated with superior AD symptom control as seen with other allergic diseases.^4^ Medical oversight from a physician with advanced knowledge is likely beneficial, especially for those with more severe disease facing flares and complex daily treatment regimens. Notably, adult patients with the greatest AD severity and anxiety/depression received specialist care, highlighting an opportunity for specialists to address the significant mental health burden of adults with more severe AD. Second, decreased symptom control predicted worse anxiety/depression in adult patients, a relationship that may be bidirectional. While greater physical symptoms logically increase patient anxiety/depression, mental detriments may hinder symptom control (ie, by affecting medication adherence); addressing mental health may be a critical intervention.^5^

Our study is limited by the use of an internet convenience sample, which may have recruited respondents experiencing a greater-than-average impact of AD. Further, we could not assess the impact of health care visit frequency on symptom control since frequency of provider visits was not queried. Finally, our survey did not receive age for a quarter of child patients, which may limit accuracy of the mean child age reported.

In conclusion, specialist care for AD among US adults is associated with superior symptom control, and specialists have a critical opportunity to address both the physical and mental impacts of AD for patients who suffer the most. Further research is needed for mental health strategies in AD and to assess if better mental health may also benefit AD symptom control.

## Conflicts of interest

Dr Schwartz serves as a paid consultant to Global Parents for Eczema Research. Author Smith Begolka has received advisory board honoraria from Amgen and Pfizer, research grants from Pfizer, and is an employee of the National Eczema Association. Author Tullos does contract work for the International Topical Steroid Awareness Network (ITSAN). Author Capozza is an employee of Global Parents for Eczema Research and has served on an Advisory Board for Incyte, Amgen, Pfizer, and LEO Pharma. Author Chong has no conflicts of interest to declare.
